# Psychological Impact of COVID-19 Pandemic on Healthcare Workers in Riyadh, Saudi Arabia: Perceived Stress Scale Measures

**DOI:** 10.1007/s44197-021-00014-4

**Published:** 2021-11-22

**Authors:** Nuha Alwaqdani, Hala A. Amer, Reem Alwaqdani, Fahad AlMansour, Hind A. Alzoman, Amal Saadallah, Salem Alsuwaidan, Barbra M. Soule, Ziad A. Memish

**Affiliations:** 1 Riyadh Dental Center, King Saud Medical City, Riyadh, Saudi Arabia; 2 Prevention and Control of Infection Administration, King Saud Medical City, Riyadh, Saudi Arabia; 3 Community Medicine Research Department, National Research Center, Cairo, Egypt; 4 Dental Private Clinics, King Saud Medical City, Riyadh, Saudi Arabia; 5 Eradah Medical Complex, Riyadh, Saudi Arabia; 6 King Faisal Specialist Hospital and Research Center, Riyadh, Saudi Arabia; 7 Research and Innovation Center, King Saud Medical City, Ministry of Health, Riyadh, Saudi Arabia; 8 Joint Commission Resources/Joint Commission International, Six Sigma Yellow Belt, Chicago, USA; 9 College of Medicine, Al-Faisal University, Riyadh, Saudi Arabia; 10 Hubert Department of Global Health, Rollins School of Public Health, Emory University, Atlanta, GA 30322 USA

**Keywords:** Psychological stress, COVID-19 pandemic, Healthcare workers

## Abstract

**Background:**

The COVID-19 pandemic has been stressful and of considerable concern among health care workers (HCWs). Being particularly at increased risk for exposure, HCWs worry about becoming infected as well as infecting co-workers, patients and family members. Such distress and panic may have destructive effects on individuals and may last long after the pandemic situation leading to depression or post-traumatic stress disorder. Therefore, the aim of the current study is to measure and investigate the prevalence of the factors affecting psychological stress during the COVID-19 pandemic among HCWs.

**Methods:**

A self-administered online survey—including perceived stress scale (PSS) questions—was disseminated among HCWs in Riyadh, Saudi Arabia (SA) between1st June and 30th July 2020.

**Results:**

A total of 469 HCWs responded to the survey for a high response rate (93.8%). The PSS revealed that 15.8% of the respondents were suffering from high stress levels, 77.2% were suffering from moderate stress levels and 7% with low stress levels. Females and junior frontline staff reported more severe stress levels. Participation on the pandemic team shows significant impact on stress levels.

**Conclusion:**

COVID-19 pandemic has increased stress levels among HCWs and affects their psychological wellbeing. Designing programs promoting HCWs mental health are crucial and emotional and psychological support strategies should be part of every public health crisis management plan.

## Introduction

Coronavirus disease 2019 (COVID-19) is a zoonotic infection caused by severe acute respiratory syndrome coronavirus 2 (SARS-CoV-2) [1]. They are enveloped, non-segmented RNA viruses that belong to the family Coronaviridae, the order Nidovirale and there are seven different strains [2]. COVID-19 was first identified in December 2019 in Wuhan, China and since has spread to all six World Health Organization (WHO) regions [3]. The WHO declared COVID-19 outbreak as a Public Health Emergency of International Concern (PHEIC) on 30th January 2020. As of November 2021, 254,847,065 million cases have been reported with more than 5,120,712 million deaths [4].

There are mental health effects that usually emerge from pandemics. The fast global spread of COVID-19 led to substantial anxiety and distress among health care workers (HCWs) as among other sectors of society [5]. The HCWs concerns are justified considering other emerging respiratory coronavirus outbreaks, such as Middle East Respiratory Syndrome Coronavirus (MERS-CoV) and SARS-CoV-1, where HCWs made up around a quarter of those infected [5].

It is understandable that HCWs are particularly at increased risk for infection as they are the ones caring for infected patients with exposure to biological samples and the patient environment. HCWs worry not only about becoming infected but also worry about infecting co-workers, patients and family members [6]. HCWs exposed to the SARS outbreak in 2002–2003 experienced high levels of psychological stress due to quarantine, being subjective to daily body temperature surveillance, and being mandated to immediately report any clinical symptoms. Such distress and panic had destructive effects, and eventually ended in some incidences where the staff refusal to care for patients [7].The staff had to face and manage the unpredictability of their work schedule, which necessitated them to adjust their private and social lives. The consequences of stress may last long after an outbreak and may result in depression or posttraumatic stress disorder [8].

The pandemic of COVID-19 has been stressful for people in the community and health care professionals. Most of individuals know the emotional components of depression: sadness, irritability, emptiness, and exhaustion. Given certain conditions, these universal experiences take over the body and transform it, sapping motivation and disrupting sleep, appetite, and attention. A recent study hypothesized that there is some degree of linkages between lockdown/isolation/pandemic disaster on stress and mental health [9].

The current view of researchers is that psychological disturbances are rising in general in medical health professionals along with auxiliary staff where all consequently suffer. Anxiety and depression often occur concurrently. Many people who experience one will be affected with the other at some point in their life. There is no doubt that the COVID-19 lockdown situation has led to significant stress with high rates of depression and anxiety. These feelings can lead to emotional, psychological, and sometimes physical hardships for individuals.

Based on governmental rules in the Kingdom of Saudi Arabia (KSA), the lockdown started partially on March 9, 2020 within the week of the first case confirmation. Later, a 24-h curfew in the main KSA cities including Riyadh started on April 6, 2020. The lockdown restriction had been eased gradually by decreasing the curfew times started on May 28, 2020 and continued in three phases, the last phase started on June 21, 2020. Our study has been conducted within those return to normality phases during June–July 2021.

Examining the literatures throughout the conduct of our study, there were few inputs relevant to the psychological impact of the COVID-19 pandemic on HCWs in the KSA. The primary objectives of this study were to measure the prevalence of psychological stress among HCWs during COVID-19 pandemic, and to investigate factors affecting the psychological stress levels. This study outcomes are likely to support the need for establishing mental health programs fitting HCWs. Such programs will assist specifically in preventing and combating the psychological stress related to pandemics and crisis situations.

## Materials and Methods

The present cross-sectional study was performed in the Riyadh region of KSA between June 1st 2020 and July 30th 2020. The target population were HCWs working in Riyadh region encompassing governmental and private hospitals. A convenience sampling technique was used to recruit the participants in the study to achieve a sample of 500 participants and their responses. Data were collected using an online questionnaire.

The participants were asked to record the following information: demographic data including gender, age, nationality, job category, qualifications, type of institution, information about role in pandemic management, such as status and duration of participation in corona pandemic team, personal experience of being suspected of or acquiring COVID-19 infection, and any underlying psychological condition and history of visit to psychiatrist.

The perceived stress scale (PSS) was used to assess feelings about the uncontrollability and unpredictability of one’s life, how often one has dealt with irritating hassles, how much change has occurred in one’s life, and confidence in one’s ability to deal with problems or difficulties in the last month. The scale was used to measure how an individual feel about the general stressfulness of their life and their ability to handle such stress. The frequency of such emotions has been gauged in our study using a questionnaire particularly the PSS [10].

PSS score is determined by following these directions: first, scores for questions 4, 5, 7, and 8 are reversed using these changes such as 0 = 4, 1 = 3, 2 = 2, 3 = 1, 4 = 0 as these questions were positively stated queries and second the adding up all the scores to get a final score. PSS was administered to six examiners. A content validation ratio (CVR) was used to determine interrater reliability. The CVR was significant for all items included under the PSS questionnaire. The CVR was 0.930 for 6 examiners (*P* < 0.05).

The PSS content details used is as follows:

**Table:** 

Query number	Query details	Never	Almost never	Sometimes	Fairly often	Very often
1	In the last month, how often have you been upset because of something that happened unexpectedly?	0	1	2	3	4
2	In the last month, how often have you felt that you were unable to control the important things in your life?	0	1	2	3	4
3	In the last month, how often have you felt nervous and “stressed”?	0	1	2	3	4
4	In the last month, how often have you felt confident about your ability to handle your personal problems?	0	1	2	3	4
5	In the last month, how often have you felt that things were going your way?	0	1	2	3	4
6	In the last month, how often have you found that you could not cope with all the things that you had to do?	0	1	2	3	4
7	In the last month, how often have you been able to control irritations in your life?	0	1	2	3	4
8	In the last month, how often have you felt that you were on top of things?	0	1	2	3	4
9	In the last month, how often have you been angered because of things that were outside of your control?	0	1	2	3	4
10	In the last month, how often have you felt difficulties were piling up so high that you could not overcome them?	0	1	2	3	4

Statistical analysis: SPSS IBM V20 (SPSS, Inc., Chicago, IL, USA) was used to analyze the collected data. A statistical significance was set at *P* < 0.05 for all the tests. Summary statistics for all variables were calculated. The summative analysis was used to encapsulate the scores from the PSS Inventory. The ANOVA test was used to establish the difference between HCW groups, such as Doctors vs. Nurses, Nurses vs. Allied Health Professionals and Doctors vs. Allied Health Professionals. The Fisher’s Exact test was used for nominal variables. To predict the outcome factor(s) from demographic data modifying the stress score, logistic regression analyses were performed, adjusting for HCWs gender, qualifications, anxiety toward acquiring COVID-19 and social avoidance. Ethical approval for the study was obtained from Institutional Review Board, King Saud Medical City.

## Results

A total of 469 HCWs responded to the survey for a high response rate (93.8%). The Perceived Stress Scale revealed that 15.8% of the respondents were suffering from high stress levels, 77.2% were suffering from moderate stress levels and 7% with low stress levels. Several frequency distributions of participant response are shown in the graphs below.

Table 1 describes the socio-demographic characteristics of the participants in the study population which explored 15 variables. The demographics showed that among the participants, there were more female subjects than male (53.7% Vs. 46.3%), the age group 25–35 years was the most predominant (55%), and the majority of study subjects were Saudi nationals (91.5%). Nurses and allied health participants composed fewer subjects than physicians and administrative staff who composed just over 70% of the participants. The majority of participants were working in governmental institutions while less participation from 1ry healthcare centers and private hospitals (74.4%, 15.8%, and 9.8% respectively).

**Table 1:** Socio-demographic characteristics of study population

Variable	Responses	Frequency (*n* = 469)	Percentage (%)
Gender	Male	217	46.3
	Female	252	53.7
Age in years	25–35	258	55
	36–45	144	30.7
	46 and above	67	14.3
Nationality	Saudi	429	91.5
	Non-Saudi	40	8.5
Highest qualification	Bachelors	221	47.1
	Masters	87	18.6
	PhD	68	14.5
	Diploma	93	19.8
Occupation	**Physicians**	**165**	**35.2**
	Consultant	67	14.3
	Specialist	50	10.2
	Resident	46	9.8
	Internship	2	.4
	**Nurses**	**82**	**17.5**
	Specialist Nurse	28	6.0
	Nurse technician	54	11.5
	**Allied health**	**54**	**11.5**
	Pharmacist	18	3.8
	Respiratory therapist technician	12	2.6
	Physiotherapy specialist technician	1	.2
	Radiology specialist technician	4	.9
	Lab technician	20	4.3
	**Admin/others**	**168**	**35.8**
Type of health institution	Primary healthcare center	74	15.8
	Government Hospital	349	74.4
	Private Hospital	46	9.8
Participated on corona pandemic team	Yes	309	65.9
	No	160	34.1
Time spent on the corona pandemic team	Less than 1 month	27	5.8
	1 month to 2 months	61	13.0
	more than 2 months	233	49.7
	Never	148	31.6
Directly involved in care of COVID 19 patients	Yes	145	30.9
	No	324	69.1
Suspected for COVID-19	Yes	170	36.2
	No	299	63.8
Confirmed for COVID-19	Yes	102	21.7
	No	367	78.3
Ever been in quarantine	10–14 days	170	36.2
	> 14 days	36	7.7
	Not at all	263	56.1
Place of quarantine	Home	170	36.2
	Dormitory	13	2.8
	Hotel or other dedicated quarantine	5	1.1
	Hospital	29	6.2
	Never been isolated	252	53.7
Ever suffered from psychological problems	Yes	46	9.8
	No	375	80.0
	May be	47	10.0
Frequency of visits to psychiatrist	Once in a month	11	2.3
	Twice a month	23	4.9
	Never	435	92.8

Almost two third of the study subjects had been involved with the pandemic team and half spent 2 months or more with the team. 69.1% of participants were not directly involved in patient care. In relation to COVID, 36.2% of the study subjects had been suspected of having COVID-19 and 21.7% of those had acquired COVID-19 infection. 44% of participants had been placed in quarantine, which was mostly at their private homes. The majority of participants did not admit to ever having had any psychological ailment or having ever visited a psychiatrist.

Table 2 shows the component loadings of the 10 variables on the two factors extracted, which are the correlations between the variable and the component (values range: − 1 to 1). The higher the absolute value of the loading, the more the factor contributes to the stress level as a variable (we have extracted 2 variables wherein the 10 items are divided into 3 variables according to the most important items, with similar responses in component 1 and simultaneously in component 2). The gap (empty spaces) on the table represents loadings that are less than 0.5. All loadings less than 0.5 were suppressed.

**Table 2:** Factors analysis for PSS queries

Component Matrix^a*^ for Perceived stress scale		
Queries	Component	
	Factor 1	Factor 2
In the last month, how often have you been upset because of something that happened unexpectedly?	.702	− .323
In the last month, how often have you felt that you were unable to control the important things in your life?	.833	
In the last month, how often have you felt nervous and “stressed”?	.760	− .367
In the last month, how often have you felt confident about your ability to handle your personal problems?	.465	.612
In the last month, how often have you felt that things were going your way?	.602	.522
In the last month, how often have you found that you could not cope with all the things that you had to do?	.657	− .307
In the last month, how often have you been able to control irritations in your life?	.522	.640
In the last month, how often have you felt that you were on top of things?	.615	.654
In the last month, how often have you been angered because of things that were outside of your control?	.640	− .413
In the last month, how often have you felt difficulties were piling up so high that you could not overcome them?	.813	

^*^a: Two components extracted. Extraction method: principal component analysis

As shown in Table 3, the percentage of participants that were categorized with stress levels differs significantly by gender and age. Participation on the Corona Team and frequency of visits to a psychiatrist were also significantly related to stress levels. The relationship between the educational qualification and stress level was not significant.

**Table 3:** Participant characteristics in relation to reported stress level as per perceived stress scale

Variables	Categories	Perceived Stress Scale stress level Total participants (469)			*P* value
		Mild 33 (7%)	Moderate 362 (77.2%)	Severe 74 (15.8%)	
Gender	Male	15 (45.5%)	167 (46.1%)	35 (47.3%)	0.000
	Female	18 (54.5%)	195 (53.9%)	39 (52.7%)	
Age	25–35	21 (4.4%)	201 (42.8%)	36 (48.6%)	0.000
	36–45	11 (2.3%)	106 (22.6%)	27 (36.5%)	
	46 and above	1 (.2%)	55 (5.7%)	11 (14.9%)	
Highest qualification	Bachelors	12 (36.4%)	173 (47.8%)	36 (48.6%)	0.15
	Masters	4 (12.1%)	67 (18.5%)	16 (21.6%)	
	PhD	7 (21.2%)	49 (13.5%)	12 (16.3%)	
	Diploma	10 (30.3%)	73 (20.2%)	10 (13.5%)	
Participation in corona pandemic team	Yes	16 (48.5%)	234 (64.6%)	59 (79.7%)	0.001
	No	17 (51.5%)	128 (35.4%)	15 (20.3%)	
Frequency of visits to psychiatrist	Once in a month	0 (0%)	10 (2.8%)	1 (1.4%)	0.000
	Twice a month	1 (3%)	17 (4.7%)	5 (6.8%)	
	Never	32 (97%)	335 (92.5%)	68 (91.9%)	

A multinomial regression analysis is presented in Table 4 using low stress level as a reference for the data obtained from the PSS. The Odds ratio (OR = 19.25, CI 2.3) demonstrates that participation on the pandemic team has a high impact on stress levels as observed in both the moderate and high stress group of respondents.

**Table 4:** Multinomial regression analysis

Perceived Stress ScaleaVa	Response	Moderate					High				
		Std. Error	*P* value	Odd ratio	95% Confidence Interval for Exp(B)		Std. Error	*P* value	Odd ratio	95% Confidence Interval for Exp(B)	
					Lower Bound	Upper Bound				Lower Bound	Upper Bound
Gender	Male	.569	.849	1.115	.365	3.402	.659	.392	1.758	.483	6.393
	Female (CR)										
Age	25–30	1.311	.839	1.304	.100	17.020	1.485	.944	.902	.049	16.562
	31–35	1.237	.477	.415	.037	4.685	1.480	.032	.042	.002	.758
	36–40	1.306	.774	.687	.053	8.890	1.512	.458	.326	.017	6.312
	41–45	1.319	.475	.390	.029	5.167	1.512	.317	.220	.011	4.259
	46–50	1764.041	.993	792	.000	.^c^	1764.042	.994	637	.000	.^c^
	> 50 (CR)										
Nationality	Saudi	2611.894	.982	8.12	.000	.^c^	2611.894	.982	2.831	.000	.^c^
	Non-Saudi (CR)										
Qualification	Bachelors	.762	.587	.661	.148	2.944	.933	.388	.447	.072	2.779
	Masters	1.090	.811	.770	.091	6.526	1.305	.358	.302	.023	3.890
	PhD	2168.758	.982	4.38	.000	.^c^	2168.758	.982	4.61	.000	.^c^
	Diploma (CR)										
Occupation	Consultant	2168.758	.982	221	.000	.^c^	2168.758	.982	190	.000	.^c^
	Specialist	1.234	.479	2.398	.213	26.957	1.405	.069	12.88	.821	202.326
	Resident	.885	.384	2.162	.382	12.244	1.075	.213	3.811	.463	31.347
	Internship	9790.523	.999	943	.000	.^c^	13,539.990	1.000	16.982	.000	.^c^
	Nurse technician	2828.649	.995	533	.000	.^c^	2828.649	.994	699	.000	.^c^
	Specialist nurse	.869	.975	.973	.177	5.345	1.265	.363	.316	.026	3.774
	Pharmacist	3602.542	.986	11,675	.000	.^c^	3602.542	.985	598	.000	.^c^
	Respiratory therapist	1218.700	.992	9686	.000	.^c^	1218.700	.992	292	.000	.^c^
	Physiotherapist	.000		100	1001	1001	.000		.214	.214	.214
	Radiologist	7962.518	.999	233	.000	.^c^	7962.518	.999	230	.000	.^c^
	Lab technician	1.086	.873	1.189	.141	10.003	1.291	.249	4.435	.353	55.730
	Receptionist	.974	.992	1.009	.150	6.809	1.279	.753	1.494	.122	18.309
	Others (CR)										
Organization	Primary health care	1097.728	.989	251	.000	.^c^	1097.728	.988	127	.000	.^c^
	Governmental hospital	1.054	.426	.432	.055	3.409	1.197	.942	1.091	.104	11.410
	Private hospital (CR)										
Participation in Corona team	Yes	1.064	.005	19.258	2.393	155.012	1.217	.000	2.050	.189	22.269
	No (CR)										
Duration of participation	< 1 month	2358.675	.995	592	.000	.^c^	2358.675	.994	364	.000	.^c^
	1–2 months	1.362	.001	.011	.001	.159	1.574	.388	.257	.012	5.622
	> 2 months	.996	.045	.135	.019	.953	1.199	.519	2.165	.207	22.688
	Never (CR)										
Involvement in patient clinical care	Yes	.681	.517	1.555	.409	5.910	.761	.145	3.031	.681	13.479
	No (CR)										
Suspected	Yes	.551	.215	1.980	.672	5.831	.636	.020	4.414	1.269	15.349
	No (CR)										
Confirmed	Yes	.734	.071	3.766	.894	15.867	.891	.551	1.700	.296	9.754
	No (CR)										
Quarantine	10–14 days	1.168	.862	1.225	.124	12.086	1.474	.895	1.216	.068	21.843
	> 14 days	1.341	.249	4.691	.339	64.976	1.745	.127	14.302	.467	437.610
	Never (CR)										
Place of quarantine	Home	1.207	.538	.475	.045	5.064	1.507	.690	.548	.029	10.516
	Dormitory	4824.474	.997	955	.000	.^c^	4824.474	.997	187	.000	.^c^
	Hotel	6634.797	.998	198	.000	.^c^	6634.797	.998	282	.000	.^c^
	Hospital	1.336	.089	.103	.007	1.413	1.910	.199	.086	.002	3.636
	Never isolated (CR)										
Psychological problem	Yes	1.712	.487	.304	.011	8.720	1.819	.945	1.133	.032	40.037
	No	1.178	.122	.162	.016	1.625	1.290	.131	.143	.011	1.790
	May be (CR)										
Psychiatrist visits	Once a month	4291.788	.997	910	.000	.^c^	4291.788	.997	310	.000	.^c^
	Twice a month	1.444	.964	1.068	.063	18.122	1.569	.815	.692	.032	15.006
	Never										

*CR* considered as reference

## Discussion

The target population of this survey were HCWs working in the Riyadh region Saudi Arabia between June 1st 2020 and July 30th 2020. The factors expected to influence the stress level experienced by the HCWs during the COVID-19 pandemic studied in this research include demographic characteristics, role in pandemic management, personal experience with COVID-19 infection and underlying psychological conditions.

The PSS revealed that 15.8% of the respondents were suffering from severe stress, 77.2% were suffering from moderate stress, and 7% with mild stress. It is possible that the recorded stress level is exaggerated as most of the responses were collected during the month of June 2020, the peak of the first wave of the pandemic in Saudi Arabia. Moreover, among the severe stress level group, 79.7% reported that they had involved in the Corona Pandemic team. This percentage was significantly higher than what reported among moderate and mild stress level groups (*P* value: 0.001). Therefore, participation in the Corona pandemic team was found to significantly affect the level of the stress measured by the perceived stress scale.

As concluded in a recent USA study, the stress level in HCWs has been intensified to outstanding levels during the COVID-19 pandemic [11]. On an international scale, the scarcity of personal protective equipment (PPE) along with surge of patients that overwhelmed the healthcare infrastructure were the main triggers of exacerbation of stress among HCWs in many countries [12]. Similarly, in Saudi Arabia, the rise in the volume of cases initially challenged the healthcare system preparedness, in spite of prior experience with MERS-COV outbreaks that also affected the HCWs’ physical and psychological conditions.

Psychological problems in HCWs during the COVID-19 pandemic in China have been studied by Que et al., who found the prevalence of anxiety, depression, insomnia, and overall psychological problems during the COVID-19 pandemic to be 46.04%, 44.37%, 28.75%, and 56.59% respectively [13]. Another study from neighboring Gulf country Oman surveyed 509 physicians and nurses using the PSS, General Anxiety Disorder (GAD) Scale, and WHO Well-Being Index and found 26% of respondents suffered from moderate to severe anxiety [14].

In another study from China by Kang et al. earlier in the SARS-CoV2 epidemic, lower levels of stress and mental health problems were reported among HCWs in comparison to our study findings. The prevalence of mild–moderate and severe disturbances was 34.4%, 22.4% and 6.2% respectively [15].

This inconsistency in the findings can be justified by the difference in the pandemic stage during which the data of each study were collected and the variance in stress levels responses among different population.

As per our study findings, severe stress level was significantly higher with younger age group, being a female and participating on the corona team. Lai et al. have reported similar findings in a study conducted to assess anxiety and depression among 1257 HCWs in 34 hospitals in China, they found that women, frontline HCWs, and those working in Wuhan, China, reported more severe degrees of all measurements of mental health symptoms than other HCWs [16].

These findings may enlighten the decision makers that a proactive approach is required to prepare HCWs to cope with stress during a crisis with more emphasis on female and junior staff. In addition, post-pandemic stress-relieving programs are required, especially for those participating on the pandemic management team.

Many recent studies which investigated the influence of COVID-19 pandemic on the mental health of HCWs have shown that nurses were more likely than attending physicians to report anxiety and to express related symptoms, such as insomnia [17–19]. The impact of this profession on the stress level was not significant in our study which could be due to nurses representing only 17.5% of our study sample. This low participation in the study may reflect that they have higher workload and were not interested in or able to respond to the survey.

Moreover, regardless of the crisis, half of all physicians are experiencing significant mental stress in the form of burnout or emotional fatigue as per a review involved published data from many countries worldwide [20]. Additionally, a cross sectional survey among 1171 randomly selected registered nurses working at USA hospitals showed that nurses experience depressive symptoms at a rate twice as high as individuals in many other professions. They are particularly vulnerable to depression given their more direct and close contact with patients [21]. Therefore, being a HCW is a predictor for significantly suffering from stress, burnout, anxiety and depression.

The majority of subjects in the different stress levels groups in our study never visited a psychiatrist. There was a significantly higher percentage among the group of those with mild stress levels who previously visited a psychiatrist compared to the groups of staff with low and high stress levels. The history of visiting a psychiatrist twice per month is significantly higher in groups with moderate and severe stress levels which may reflect that those who are keen to seek help for mental health problems may be more aware of realizing their stress. In addition, the underlying mental health condition of the individual may affect their perceived stress level.

By multinomial regression analysis, participation on the pandemic team shows significant impact on stress levels as observed in the moderate and high stress group of respondents. Moderate stress level was significantly associated with age group (31–35 years) and anyone suspected for COVID, possibly explained by fear from contracting the disease and being in quarantine until COVID-19 infection was ruled out. A survey conducted among HCWs in Saudi Arabia in October 2020 showed that HCWs who experienced isolation due to COVID-19 suspicion or infection had a higher anxiety level [17].

One of the limitations of this study, it didn’t investigate the effect of the personal factors on the HCWs stress level. Those personal factors may include home environment, social relationship and health condition. Another limitation, the collected data doesn’t allow to calculate the number participating facilities. A third limitation, the responses of the participants may vary if the survey repeated in a different period when number of COVID-19 cases dropped down and restrictions totally eased in KSA.

**Fig. 1:**
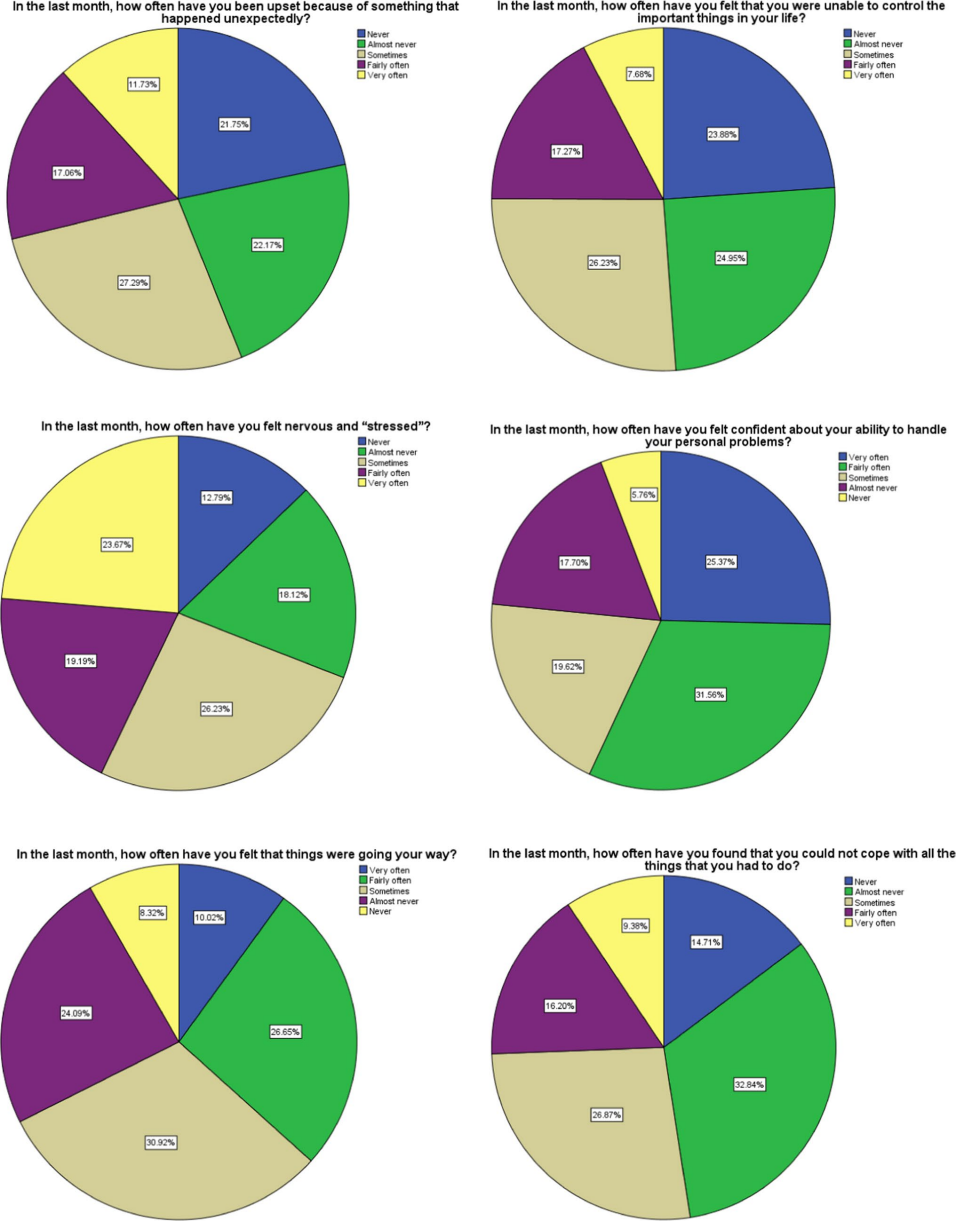

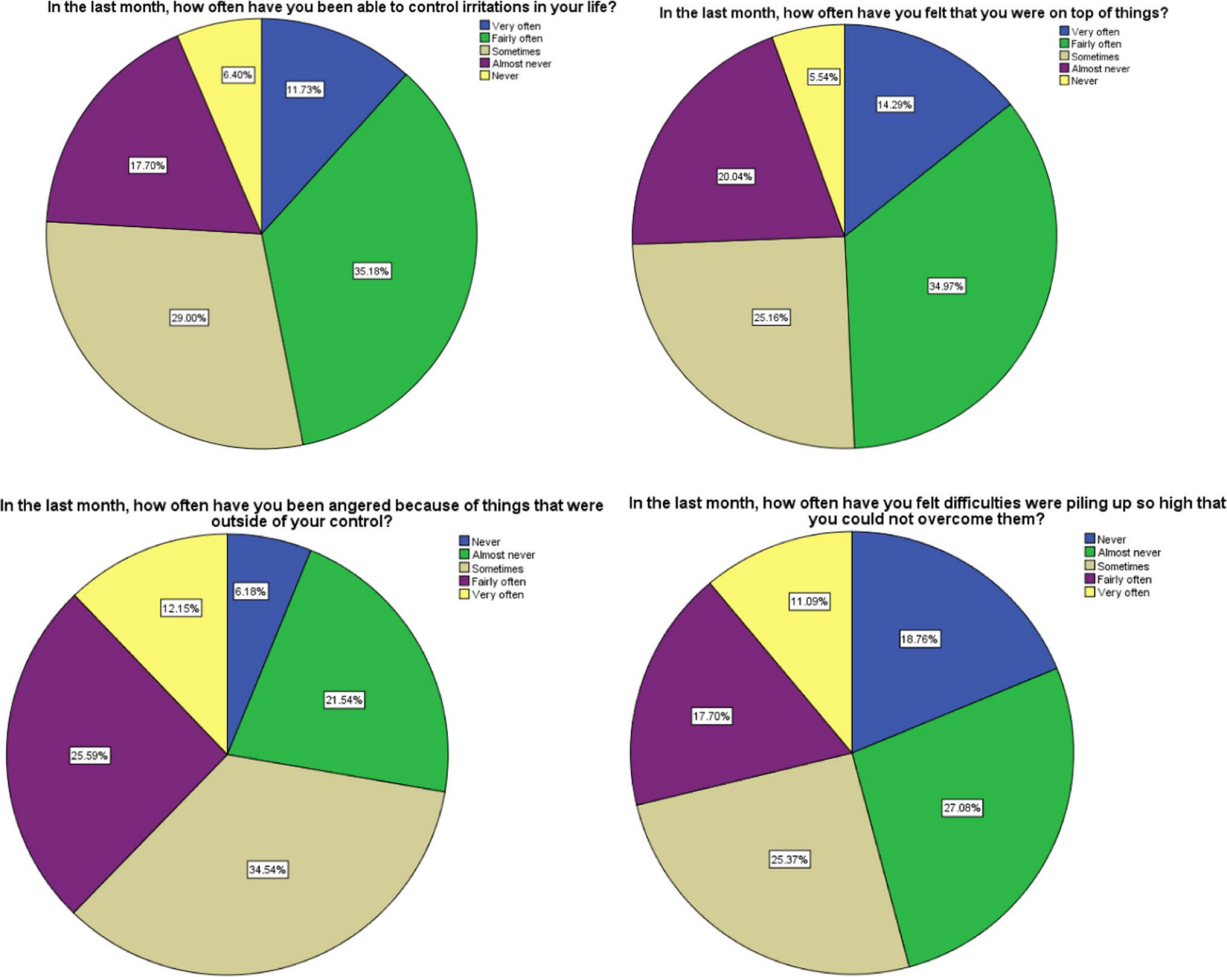
Frequency distribution of responses from perceived stress scale

In summary, our findings demonstrate that a public health crisis can increase the stress level among HCWs and affect their psychological wellbeing. Designing programs promoting HCWs mental health are crucial and emotional and psychological support strategies should be part of every public health crisis management plan and may be tailored to HCW populations according to their expected stress levels. In the COVID-19 pandemic, sustainable availability of sufficient PPE, continuous awareness and communication and accessible support for staff are key measures to reduce the HCWs’ stress. There is a need for high-quality data on the mental health effects of the COVID-19 pandemic across health care professionals that will guide the decision makers’ preparedness plans for this and future epidemics (Fig. 1).

## Declarations

### Availability of Data and Material

All study data are available on request.
